# Comparative midterm outcomes of contemporary revascularization strategies for acute lower limb ischemia: a retrospective cohort study

**DOI:** 10.3389/fcvm.2026.1963564

**Published:** 2026-09-29

**Authors:** Hua Zhang, Pengwei Guo, Baoxing Chen, Jinbao Geng, Zhengzuo Lv, Xin Shao, Huipeng Li, Guohua Wang

**Affiliations:** Department of General Surgery II (Vascular Surgery, Bariatric and Metabolic Surgery), Xinxiang Central Hospital, Xinxiang, Henan, China

**Keywords:** acute lower limb ischaemia, endovascular, hybrid procedure, open surgery, revascularization

## Abstract

**Background:**

Acute lower limb ischaemia (ALLI) is a vascular emergency associated with high morbidity and mortality. Contemporary revascularization strategies include open surgery,endovascular intervention,and hybrid approaches,yet comparative midterm outcomes remain incompletely defined.

**Methods:**

We conducted a single - center retrospective cohort study of 842 consecutive patients with ALLI treated at Xinxiang City Central Hospital between January 2019 and December 2023,with follow - up complete through May 2026.Patients underwent open surgery(*n* = 452),endovascular intervention(*n* = 198),or hybrid procedures (*n* = 192). Baseline characteristics, peri-operative outcomes, and long - term events were collected. The pre - specified primary outcome was the composite of major amputation and/or all-cause mortality at 3 years. Secondary outcomes included individual components, peri-operative mortality, acute kidney injury, and re-intervention. Multivariable Cox regression and Fine-Gray competing risk models were applied, together with propensity score inverse probability of treatment weighting (IPTW) as sensitivity analyses.

**Results:**

The mean age was 74 ± 13, 70 ± 12,and 71 ± 13 years in the open, endovascular, and hybrid groups, respectively. Endovascular treatment was associated with lower peri - operative mortality (adjusted OR 0.32,95% CI 0.15–0.68, *p* = 0.003) and reduced acute kidney injury (OR 0.48,95% CI 0.25–0.92,*p* = 0.027).Over a median follow - up of 34 months (IQR 28–41),cumulative 3 - year major amputation rates were 11.6%, 21.9%, and 13.5%,and all - cause mortality 24.1%,12.8%,and 20.3% in the open, endovascular, and hybrid groups,respectively.In adjusted competing-risk regression, endovascular treatment was associated with a lower subdistribution hazard of major amputation (sHR 0.69, 95% CI 0.49–0.97, *p* = 0.034); however, this association was model-dependent and contrasted with the higher crude amputation incidence observed in the endovascular group. Endovascular treatment was also associated with a lower risk of the composite endpoint of major amputation and/or all-cause mortality (HR 0.71, 95% CI 0.56–0.90, *p* = 0.006), whereas hybrid approaches did not differ significantly from open surgery.

**Conclusions:**

Endovascular revascularization for ALLI offers improved peri - operative safety and favorable midterm composite outcomes compared with open surgery in this observational cohort. Hybrid strategies demonstrate intermediate results. These findings support individualized treatment selection, but definitive causal inferences are limited by the non - randomized design and potential residual confounding.

## Introduction

Acute lower limb ischaemia (ALLI) remains a time-critical vascular emergency associated with substantial morbidity and mortality, with persistently high risks of major amputation and death despite advances in modern vascular care (1–4). The condition is characterised by a sudden reduction in limb perfusion, typically resulting from thrombotic or embolic arterial occlusion, and requires prompt diagnosis and urgent revascularisation to preserve limb viability and improve survival (2, 3). Recent advances in vascular diagnostic strategies, imaging techniques, and clinical risk assessment have contributed to earlier recognition and improved management of patients with acute vascular disorders (5, 6). However, even with timely intervention, outcomes remain poor, particularly in patients presenting with advanced ischaemia, multiple comorbidities, or delayed access to specialist care (1, 7). Contemporary epidemiological data indicate that ALLI continues to impose a significant clinical and economic burden, with adverse outcomes extending well beyond the initial hospitalisation period (7–9). Although ALLI commonly results from acute thrombotic or embolic events, its occurrence may also be associated with inadequate management of underlying vascular disease, delayed initiation of appropriate vascular therapy, or poor adherence to recommended preventive and medical treatments. Therefore, optimisation of vascular risk-factor control and adherence to evidence-based therapies remain important strategies for reducing the incidence and burden of ALLI.

The management of ALLI has evolved considerably over the past decades. Historically, open surgical revascularization, including embolectomy, bypass surgery, and thromboendarterectomy has been regarded as the cornerstone of treatment, particularly in patients with immediately threatened limbs or complex occlusive disease (2, 10). While these approaches remain highly effective in selected cases, they are also associated with considerable peri-operative risk, especially in elderly or comorbid patients. In parallel, the rapid development of endovascular techniques, including catheter-directed thrombolysis, mechanical thrombectomy, and percutaneous revascularisation, has expanded the therapeutic landscape (3, 4, 10, 11). Furthermore, hybrid procedures combining open and endovascular components have emerged as a versatile strategy to address complex anatomical patterns of disease while potentially minimising procedural invasiveness (10–12).

These contemporary revascularisation strategies offer several theoretical and practical advantages. Endovascular and hybrid approaches may reduce physiological stress, shorten hospital stay, and improve tolerability in high-risk populations, while maintaining acceptable technical success and limb salvage rates (3, 4, 11, 12). As a result, there has been a progressive shift towards less invasive treatment paradigms in many vascular centres. Nevertheless, this evolution has not been accompanied by a proportional increase in high-quality comparative evidence, and the optimal treatment strategy for ALLI remains uncertain.

The current evidence base is limited and heterogeneous. Since the landmark randomised trials of the 1990s, which primarily compared surgical revascularisation with thrombolysis, there has been a relative paucity of contemporary randomised data evaluating modern endovascular and hybrid techniques (9, 13). More recent observational studies and registry-based analyses have provided valuable insights, but their findings are inconsistent. Some large-scale national and multicentre studies suggest improved early outcomes or amputation-free survival with endovascular approaches, whereas others report comparable or even favourable outcomes with open surgery (11, 12, 14, 15). Importantly, many of these studies are limited by selection bias, variation in treatment allocation, and inadequate adjustment for key confounders such as the severity of ischaemia at presentation, which remains one of the strongest predictors of outcome in ALLI (8, 11, 14).

Another critical limitation in the literature is the lack of standardised outcome reporting. Variability in definitions of clinical endpoints, differences in follow-up duration, and inconsistent reporting of procedural details make meaningful comparisons between studies challenging (9, 16). In addition, many reports focus predominantly on short-term or peri-operative outcomes, whereas data on midterm outcomes, particularly from multicentre, real-world settings remain relatively scarce (11, 12, 14, 15, 17). This gap in evidence limits the ability to optimise patient selection, refine treatment strategies, and develop robust, evidence-based clinical guidelines.

Given these limitations, there is a clear need for contemporary, real-world data evaluating the comparative effectiveness of open, endovascular, and hybrid revascularisation strategies in patients with ALLI. Such data are essential not only to inform clinical decision-making and patient counselling but also to support future research, including the design of adequately powered prospective trials. Therefore, this study aims to evaluate peri-operative and midterm outcomes following open, endovascular, and hybrid revascularisation for acute lower limb ischaemia, and to compare the risks of major amputation, death, and re-intervention among these treatment strategies after adjustment for important baseline differences.

## Methods

### Study design and setting

This investigation was conducted as a retrospective observational cohort study at Xinxiang City Central Hospital, China, a tertiary referral centre with established experience in arterial disease treatment. The study was reported in accordance with the Strengthening the Reporting of Observational Studies in Epidemiology (STROBE) statement (16). Ethical approval was obtained from the institutional review board (IRB No. 2025 - 021) on 10 January 2025, covering retrospective analysis of all anonymised data collected from January 2019 onward; the requirement for individual informed consent was formally waived.

### Study population

All consecutive adult patients (age >18 years) presenting with symptomatic ALLI within 14 days of symptom onset and treated by revascularisation between January 2019 and December 2023 were considered. The final data lock for all clinical events was set at 31 May 2026, ensuring a minimum follow - up of 29 months for the last enrolled patient. Exclusion criteria were: conservative management without revascularisation; primary major amputation without revascularisation attempt; traumatic or non - occlusive causes; ischaemia secondary to popliteal artery aneurysm; and incomplete clinical or follow - up data. Patients with Rutherford III ischaemia were not excluded because the study aimed to evaluate contemporary revascularisation strategies among patients undergoing attempted treatment in routine clinical practice. However, we acknowledge that Rutherford III represents irreversible limb ischaemia, for which primary amputation may be appropriate in selected cases. Therefore, a sensitivity analysis excluding Rutherford III patients was performed to evaluate the robustness of the findings. A total of 1,284 patients were screened, of whom 442 were excluded, leaving 842 patients for analysis (Figure 1).

**Figure 1:**
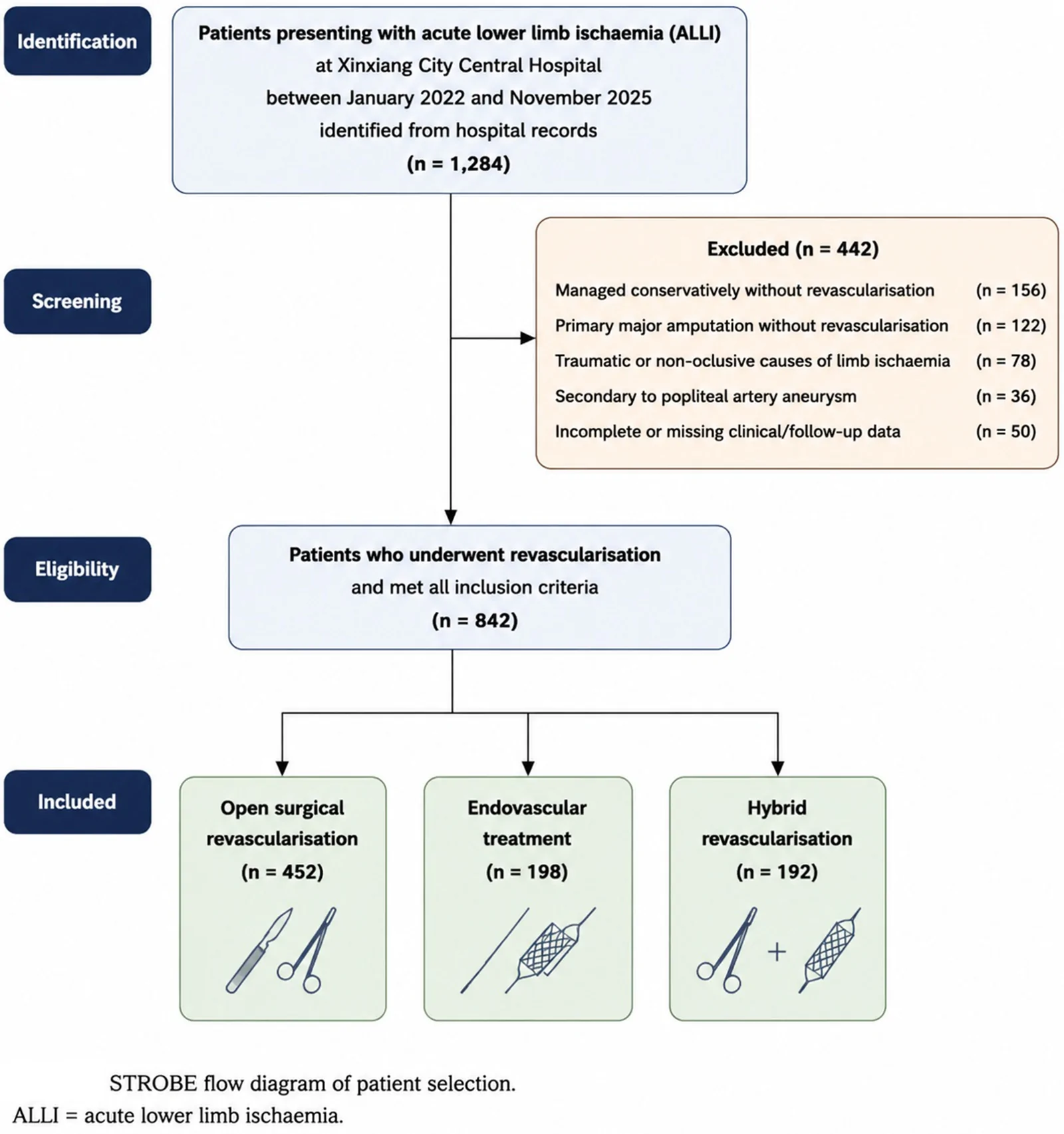
Strobe flow diagram of patient selection. A total of 1,284 ALLI patients were identified between January 2019 and December 2023; 442 were excluded. The final cohort included 842 patients who underwent revascularisation (open: 452, endovascular: 198, hybrid: 192).

### Data collection and definitions

Data were extracted from electronic medical records using a standardised case report form. Baseline variables included age, sex, Rutherford classification, cardiovascular risk factors, comorbidities, and medication history. Procedural details and peri - operative events were recorded. Follow - up data included death, major amputation (above - ankle), re - intervention (any clinically driven repeat procedure on the index limb), and acute kidney injury (KDIGO criteria) (17). Technical success was defined as completion of the intended procedure with restored inline flow to the foot without unplanned conversion. To avoid immortal time bias, all time - to - event analyses were calculated from the date of hospital presentation (index procedure date) to the date of event or last follow - up.

### Treatment groups

Patients were stratified by the initial revascularisation strategy: open surgery (thromboembolectomy, bypass, or endarterectomy), endovascular (angioplasty, stenting, thrombolysis, or mechanical thrombectomy), or hybrid (combined open and endovascular techniques in the same episode). The choice was made by the treating team based on clinical and anatomical factors, reflecting real - world practice.

### Outcomes

The pre - specified primary outcome was the composite of major amputation and/or all - cause mortality at 3 years after the index procedure. Secondary outcomes included peri - operative mortality (within 30 days or during index admission), acute kidney injury, re - intervention, and individual components of the primary endpoint at 1, 2, and 3 years as exploratory analyses.

## Statistical analysis

Continuous variables were expressed as mean ± SD or median (IQR), categorical as n (%). Comparisons used ANOVA, Kruskal - Wallis, or chi - square tests as appropriate.

Multivariable Cox proportional hazards models were used for the composite endpoint and for mortality. For major amputation, because death is a competing risk, we used Fine - Gray subdistribution hazard models as the primary analysis. Covariates included age, sex, Rutherford stage, smoking, diabetes, hypertension, atrial fibrillation, chronic kidney disease, and embolic presentation. Given the strong association between Rutherford stage and treatment selection, we performed two sensitivity models: Model 1 adjusted for all covariates including Rutherford stage; Model 2 excluded Rutherford stage to avoid over - adjustment. Results from both models are presented in the Supplementary Material, and the primary models presented in the main text include Rutherford stage.

Propensity score inverse probability of treatment weighting (IPTW) was performed as a secondary sensitivity analysis to further reduce selection bias. Propensity scores for endovascular versus open surgery were estimated using logistic regression including all baseline covariates. Stabilised weights were applied, and covariate balance was assessed by standardised mean differences (SMD <0.10 after weighting) (Supplementary Table S1). Weighted Cox and logistic regressions were repeated.

All analyses were performed on the basis of the actual follow - up available up to 31 May 2026. Patients who had not reached the 3 - year time point were censored at their last follow - up date. Two - sided *p* < 0.05 was considered significant. Analyses used R 4.3.0 (R Foundation) and SPSS 26.0.

## Results

### Study population and baseline characteristics

A total of 842 patients were included (open: 452, endovascular: 198, hybrid: 192). Baseline characteristics are shown in Table 1. Patients undergoing open surgery were older and more often had Rutherford IIb - III ischaemia, while endovascular and hybrid groups had higher rates of smoking and dyslipidaemia. Atrial fibrillation and chronic kidney disease were more frequent in the open group. The median (IQR) follow - up duration was 34 (28–41) months. Complete 3 - year follow - up was available for 712 patients (84.6%); the remainder were censored due to death or loss to follow - up. Because Rutherford III ischaemia represents irreversible limb injury and may influence treatment selection and outcomes, we performed a sensitivity analysis excluding Rutherford III patients. The direction and magnitude of the adjusted associations remained consistent, suggesting that the primary findings were not solely driven by inclusion of patients with irreversible ischaemia (Supplementary Supplementary Table S2).

**Table 1:** Baseline characteristics of the study population.

Characteristic	Open (*n* = 452)	Endovascular (*n* = 198)	Hybrid (*n* = 192)	p value
Age -y	74 ± 13	70 ± 12	71 ± 13	<0.001
Sex				0.02
Male	235 (52%)	120 (61)	115 (60)	
Female	217 (48%)	78 (39)	77 (40)	
Rutherford stage				<0.001
I	70 (15%)	50 (25%)	20 (10%)	
IIa	150 (33%)	85 (43%)	80 (42%)	
IIb	170 (38%)	50 (25%)	70 (36%)	
III	62 (14%)	13 (7%)	22 (12%)	
Acute-on-chronic arterial thrombosis (defined as acute thrombosis occurring in a pre-existing atherosclerotic vessel with clinical or imaging evidence of chronic peripheral arterial disease)	140 (31%)	100 (50%)	85 (44%)	<0.001
Arterial embolism (defined as acute arterial occlusion due to embolic material originating from a proximal cardiac or arterial source)	190 (42%)	45 (23%)	55 (29%)	<0.001
Compartment syndrome and fasciotomy	20 (4%)	10 (5%)	12 (6%)	0.44
Coronary artery disease	140 (31%)	60 (30%)	55 (29%)	0.09
Peripheral arterial disease	75 (17%)	30 (15%)	32 (17%)	<0.001
Past atrial fibrillation	180 (40%)	45 (23%)	48 (25%)	<0.001
New atrial fibrillation	40 (9%)	8 (4%)	14 (7%)	0.015
Chronic kidney disease	110 (24%)	55 (28%)	50 (26%)	0.003
Hypertension	330 (73%)	150 (76%)	132 (69%)	0.10
Diabetes	120 (27%)	60 (30%)	55 (29%)	0.48
Dyslipidaemia	240 (53%)	130 (66%)	100 (52%)	0.003
Malignancy	70 (16%)	30 (15%)	36 (19%)	0.21
Smoking history	200 (44%)	115 (58%)	120 (63%)	<0.001
Angiotensin enzyme inhibitor	135 (30%)	50 (25%)	60 (31%)	0.55
*β* blocker	180 (40%)	70 (35%)	65 (34%)	0.35
Calcium channel blocker	110 (24%)	45 (23%)	44 (23%)	0.79
Aspirin	180 (40%)	120 (61%)	85 (44%)	<0.001
Clopidogrel	50 (11%)	40 (20%)	28 (15%)	0.014
Vitamin K antagonist	45 (10%)	25 (13%)	15 (8%)	0.27
Other oral anticoagulant	75 (17%)	25 (13%)	28 (15%)	0.34
Statin	230 (51%)	120 (61%)	100 (52%)	0.043

Data are mean ± SD or n (%).

### Procedural characteristics

Procedural details are summarised in Supplementary Table S3. Open procedures included Fogarty thromboembolectomy (62%), bypass (28%), and endarterectomy (10%). Endovascular interventions comprised angioplasty/stenting (55%), catheter - directed thrombolysis (25%), and mechanical thrombectomy (20%). Hybrid procedures combined open thrombectomy with adjunctive angioplasty or stenting in 78% of cases.

### Peri - operative outcomes

Unadjusted peri - operative mortality was 4.6% (open), 1.5% (endovascular), and 3.6% (hybrid). In multivariable logistic regression (Table 2), endovascular treatment was associated with lower peri - operative mortality (OR 0.32, 95% CI 0.15–0.68, *p* = 0.003). Acute kidney injury occurred in 13.2% overall; endovascular treatment reduced its risk (OR 0.48, 95% CI 0.25–0.92, *p* = 0.027) (Table 3).

**Table 2:** Multivariable logistic regression analysis for peri-operative mortality.

Parameter	OR	95% CI	p value
Open surgery	1.0 (ref.)	—	—
Endovascular	0.32	0.15–0.68	0.003
Hybrid	0.81	0.45–1.45	0.48
Age (per year)	1.02	1.00–1.04	0.08
Rutherford stage	1.65	1.22–2.24	0.002
Chronic kidney disease	2.41	1.56–3.71	<0.001

Odds ratios (OR) with 95% confidence intervals (CI) and p values for predictors of peri-operative death. Open surgery is the reference group. Variables include treatment modality and age.

**Table 3:** Multivariable logistic regression analysis for acute kidney injury.

Parameter	OR	95% CI	p value
Open surgery	1.0 (ref.)	—	—
Endovascular	0.48	0.25–0.92	0.027
Hybrid	1.29	0.78–2.12	0.32
Rutherford stage	1.58	1.15–2.18	0.004
Chronic kidney disease	3.05	2.01–4.63	<0.001

ORs (95% CI) and p values for predictors of acute kidney injury within 30 days post-procedure. Open surgery is the reference category. Variables include treatment modality and Rutherford stage.

### Long - term outcomes

Table 4 presents the absolute numbers and cumulative incidence (Kaplan - Meier estimates) of re - intervention, major amputation, and mortality at 1, 2, and 3 years. The 3 - year cumulative mortality was 24.1% (open), 12.8% (endovascular), and 20.3% (hybrid) (Figure 2). In multivariable Cox regression, mortality did not differ significantly between groups (endovascular vs open: HR 0.88, 95% CI 0.61–1.26, *p* = 0.47) (Table 5).

**Table 4:** Absolute numbers and cumulative incidence of clinical events during follow-up.

Outcome	Open	Endovascular	Hybrid
Re-intervention			
1 year	31/452 (6.8%)	35/198 (17.5%)	29/192 (15.2%)
2 years	43/452 (9.5%)	40/198 (20.3%)	34/192 (17.8%)
3 years	46/452 (10.1%)	42/198 (21.0%)	36/192 (18.5%)
Major amputation			
1 year	40/452 (8.9%)	37/198 (18.7%)	21/192 (10.9%)
2 years	49/452 (10.8%)	47/198 (23.5%)	26/192 (13.6%)
3 years	52/452 (11.6%)	43/198 (21.9%)	26/192 (13.5%)
All - cause mortality			
Death (1 year)	97/452 (21.5%)	19/198 (9.8%)	33/192 (17.4%)
Death (2 years)	108/452 (23.8%)	22/198 (11.2%)	37/192 (19.2%)
Death (3 years)	109/452 (24.1%)	25/198 (12.8%)	39/192 (20.3%)

Absolute number of events (n/total) and cumulative incidence (%) of re-intervention, major amputation, and all-cause mortality at 1, 2, and 3 years, stratified by treatment group.

**Figure 2:**
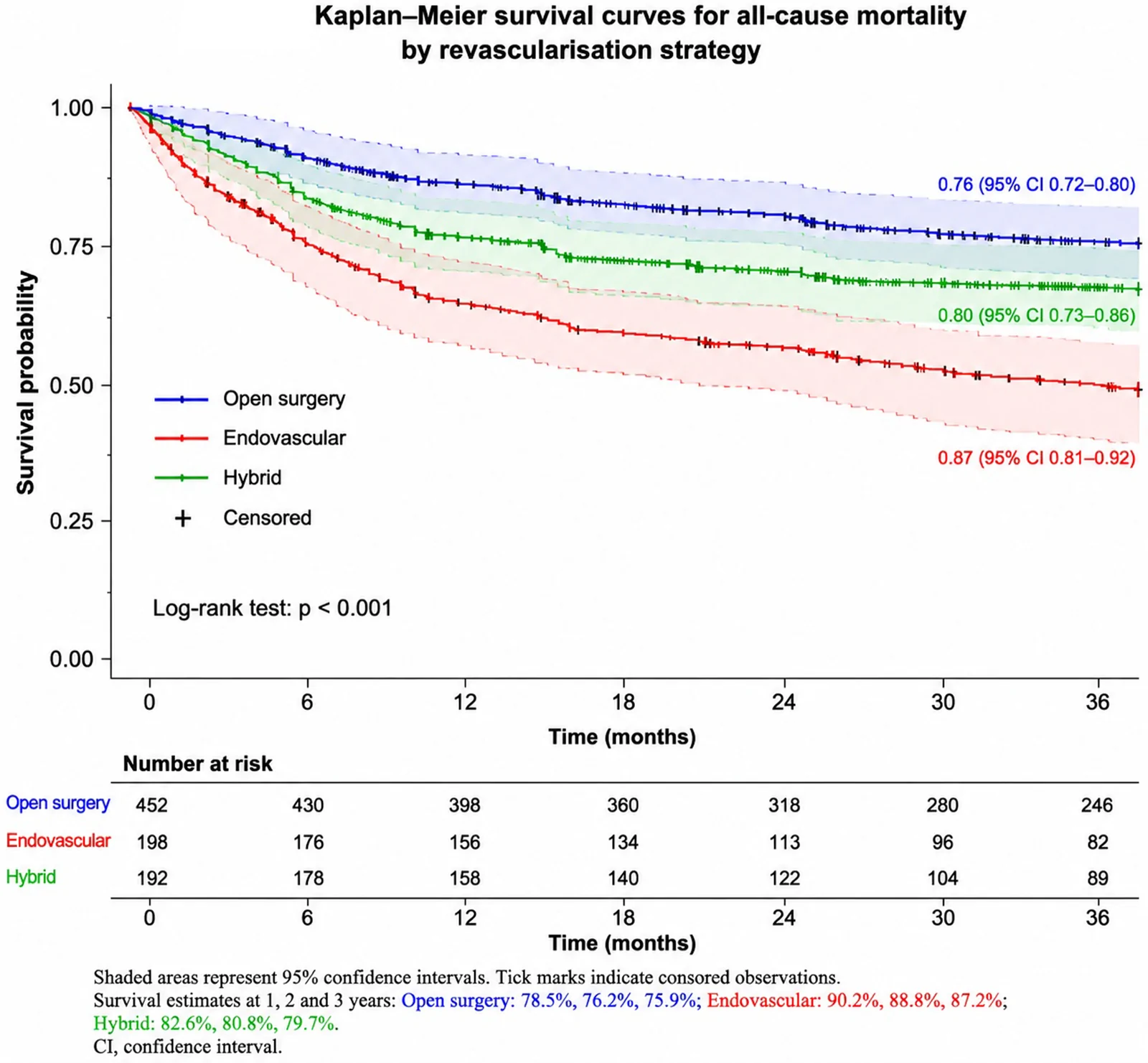
Kaplan-meier curves for overall survival over 36 months. The endovascular group shows a trend towards better survival, but the difference was not significant after adjustment.

**Table 5:** Multivariable Cox regression analysis for mortality.

Parameter	HR	95% CI	p value
Open surgery	1.0 (ref.)	—	—
Endovascular	0.88	0.61–1.26	0.47
Hybrid	0.96	0.69–1.34	0.81

HRs (95% CI) and p values for overall mortality according to revascularisation strategy, with open surgery as the reference.

For major amputation, the 3-year cumulative incidence was 11.6% (open), 21.9% (endovascular), and 13.5% (hybrid) (Figure 3). Notably, the crude incidence of major amputation was higher in the endovascular group than in the open surgery group, suggesting substantial baseline differences in patient characteristics and disease severity between treatment groups. In the primary Fine-Gray competing risk model (Table 6), endovascular treatment was associated with a lower subdistribution hazard of major amputation (sHR 0.69, 95% CI 0.49–0.97, *p* = 0.034) compared with open surgery. However, this apparent protective association was model-dependent, as the statistical significance was attenuated when Rutherford stage was excluded from the model (HR 0.75, 95% CI 0.53–1.06, *p* = 0.10). Therefore, the adjusted association should not be interpreted as definitive evidence of superior limb salvage with endovascular treatment. The hybrid group showed no significant difference (sHR 0.88, 95% CI 0.60–1.30, *p* = 0.52).

**Figure 3:**
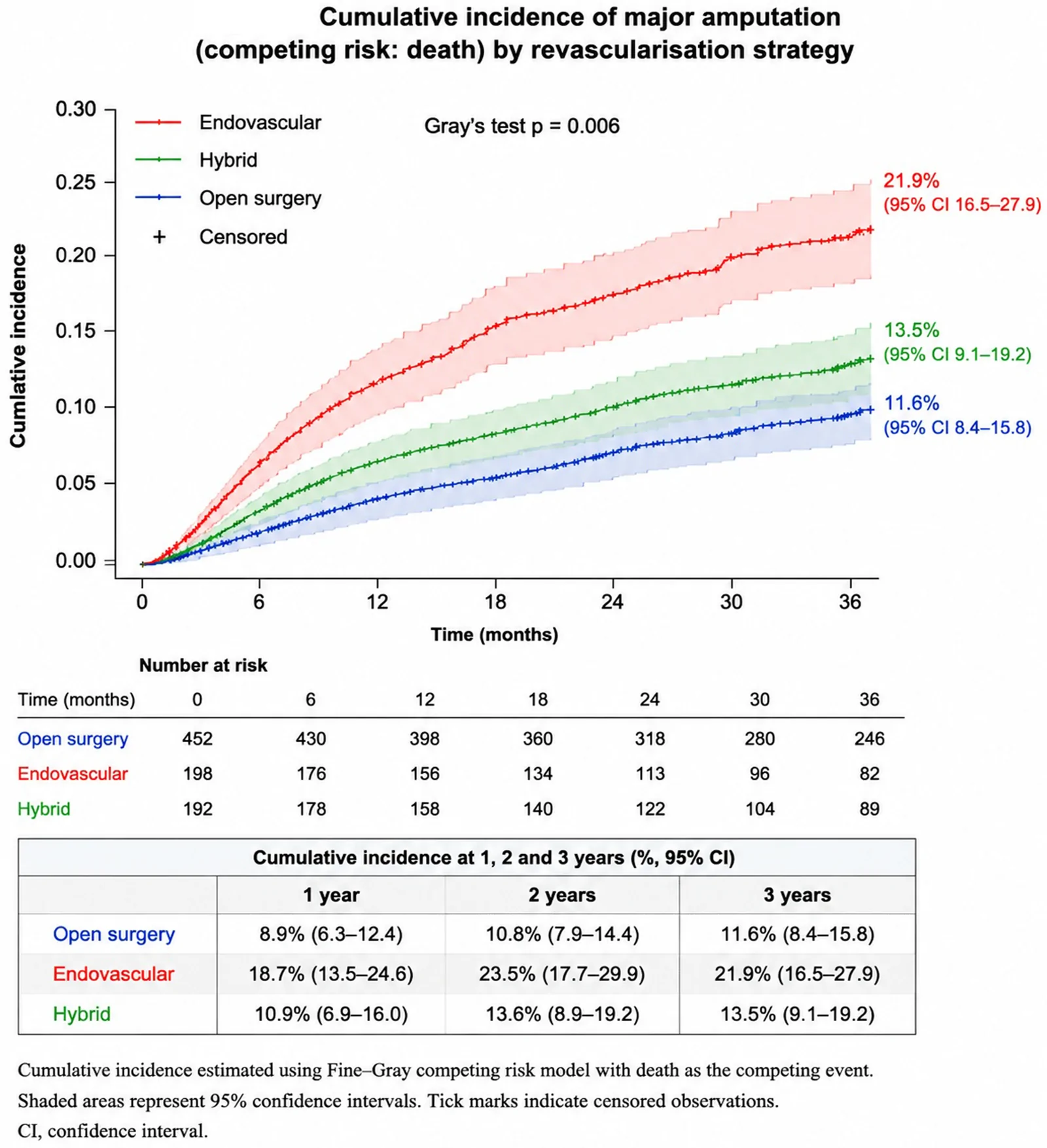
Cumulative incidence of major amputation (Fine-Gray curves). Endovascular intervention shows a lower subdistribution hazard compared with open surgery in adjusted analysis.

**Table 6:** Fine-Gray competing risk regression for major amputation (primary analysis).

Variable	sHR	95% CI	p value
Open surgery	1.0 (ref)	—	—
Endovascular	0.69	0.49–0.97	0.034
Hybrid	0.88	0.60–1.30	0.52
Rutherford stage	2.75	1.48–5.10	<0.001
Age	1.03	1.01–1.05	0.012
Diabetes	1.40	1.00–1.96	0.048
Atrial fibrillation	1.35	0.98–1.86	0.065
CKD	1.62	1.14–2.30	0.007

Multivariable Cox proportional hazards model for predictors of major amputation. Open surgery is reference category.

The composite endpoint (major amputation and/or all-cause mortality) at 3 years occurred in 30.5% of patients in the open surgery group, 20.1% in the endovascular group, and 27.4% in the hybrid group (Figure 4). Correspondingly, the 3-year event-free survival estimates were 69.5%, 79.9%, and 72.6%, respectively. In multivariable Cox regression (Table 7), endovascular treatment was associated with a lower hazard of the composite endpoint compared with open surgery (HR 0.71, 95% CI 0.56–0.90, *p* = 0.006). Hybrid procedures were not significantly different (HR 0.87, 95% CI 0.68–1.11, *p* = 0.26).

**Figure 4:**
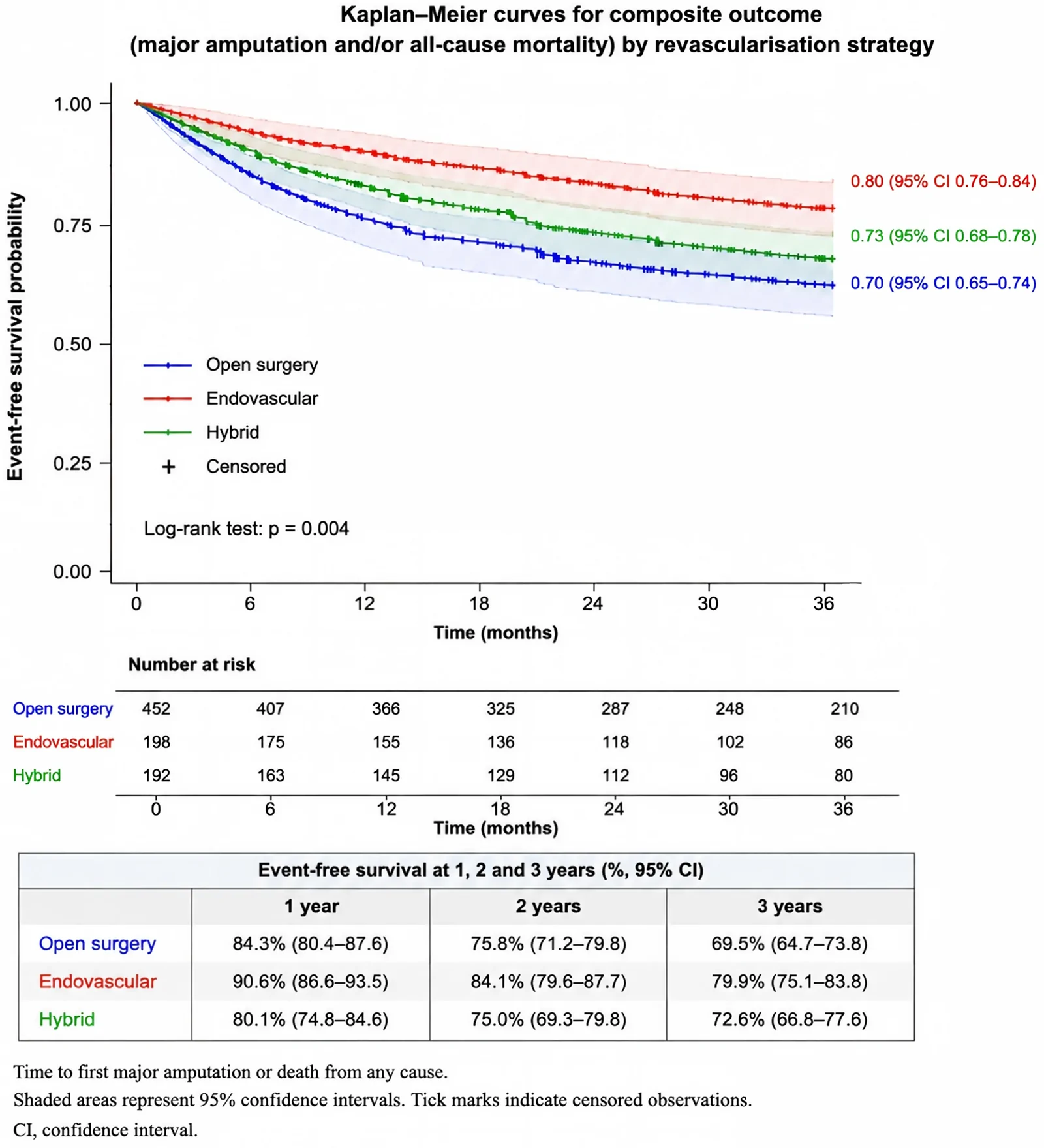
Kaplan-Meier curves for the composite outcome of major amputation and/or death. Endovascular treatment was associated with a lower risk.

**Table 7:** Multivariable Cox regression analysis for composite outcome (major amputation and/or death).

Parameter	HR	95% CI	p value
Open surgery	1.0 (ref.)	—	—
Endovascular	0.71	0.56–0.90	0.006
Hybrid	0.87	0.68–1.11	0.26

HRs (95% CI) and p values for the composite endpoint of major amputation and/or death. Open surgery is used as the reference group.

Re - intervention rates at 3 years were 10.1% (open), 21.0% (endovascular), and 18.5% (hybrid) (Table 4).

### Propensity - weighted sensitivity analyses

After IPTW, all covariates achieved SMD <0.10 (Supplementary Table S1). IPTW - adjusted analyses confirmed the direction and magnitude of the primary findings: endovascular treatment remained associated with reduced peri - operative mortality (OR 0.34, 95% CI 0.16–0.72), lower acute kidney injury (OR 0.51, 95% CI 0.27–0.96), and a lower composite endpoint (HR 0.73, 95% CI 0.58–0.92). Detailed IPTW results are shown in Supplementary Table S4.

## Discussion

This retrospective cohort study provides contemporary real - world evidence on peri - operative and midterm outcomes following open, endovascular, and hybrid revascularisation for ALLI. The principal findings demonstrate that endovascular treatment is associated with lower peri - operative mortality and reduced risk of the composite endpoint, while long - term survival appears similar across strategies. The lower crude incidence of the composite endpoint in the endovascular group was reflected by higher 3-year event-free survival compared with open surgery. However, as with all observational comparisons, this association should be interpreted cautiously because treatment allocation was influenced by clinical severity, anatomical characteristics, and physician judgement. Importantly, the observed benefit for amputation reduction was sensitive to adjustment for Rutherford stage, and the crude amputation rates were actually higher in the endovascular group, highlighting the substantial confounding by indication.

The peri - operative mortality rates (4.6% open, 1.5% endovascular) are consistent with recent large registries (9, 10, 12). The lower invasiveness of endovascular procedures likely contributes to early safety, especially in elderly patients with comorbidities. Importantly, the minimally invasive nature of contemporary endovascular therapy reflects not only advances in revascularisation techniques but also refinements in procedural practice, including ultrasound-guided vascular access and percutaneous vascular closure strategies, which may further reduce access-related complications, procedural burden, and recovery time. However, the lack of a significant long - term survival difference aligns with the understanding that ALLI outcomes are predominantly determined by underlying cardiovascular disease rather than the revascularisation modality itself (5, 13).

Our findings are broadly consistent with the recently published Acute Lower Limb Ischaemia Vascular Outcomes Evaluation (ALIVE) Registry, a multicentre retrospective cohort evaluating contemporary open, hybrid, and endovascular revascularisation strategies for ALLI (17). Similar to the ALIVE Registry, our study demonstrates that endovascular approaches are increasingly incorporated into contemporary ALLI management and may provide advantages in early peri-operative outcomes, particularly among high-risk patients. However, both studies highlight the complexity of treatment comparisons in ALLI, as treatment allocation is influenced by clinical severity, anatomical characteristics, and operator judgement. Although our cohort demonstrated a lower adjusted risk of the composite endpoint and major amputation among patients treated with endovascular strategies, these findings should be interpreted cautiously given the observational design and potential residual confounding. The consistency of findings between our single-centre experience and multicentre registry data supports the relevance of contemporary endovascular approaches, while also emphasising the need for prospective comparative studies to define optimal patient selection and treatment strategies.

The discordance between crude and adjusted amputation rates deserves careful interpretation. In unadjusted analyses, endovascular patients had higher amputation rates (21.9% vs 11.6%), but after multivariable adjustment (including Rutherford stage), the association reversed. This reversal suggests that the endovascular group initially presented with more severe ischaemia, and that adjustment may over - correct, potentially masking a true higher risk. We therefore present both adjusted and unadjusted results, and we emphasise that our findings should be considered hypothesis - generating rather than definitive. The sensitivity analysis excluding Rutherford stage from the model (HR 0.75, *p* = 0.10) further indicates that the significance is not robust across model specifications.

The higher re - intervention rates in endovascular and hybrid groups (21% and 18.5% at 3 years) are consistent with previous reports (10). However, these findings should be interpreted cautiously because the endovascular category included heterogeneous procedures, including angioplasty/stenting, catheter-directed thrombolysis, and mechanical thrombectomy, which may have different durability profiles. Differences in lesion complexity, technical approach, and use of adjunctive technologies may contribute to variation in long-term outcomes.

### Strengths and limitations

Strengths include the large, consecutive cohort, comprehensive adjustment for multiple confounders, and the use of competing - risk modelling and propensity weighting to address inherent biases of observational data.

Limitations include: (1) the retrospective, non - randomized design – treatment allocation was not controlled, and residual confounding from unmeasured factors (e.g., frailty, operator experience, detailed anatomic complexity) cannot be excluded; (2) the single - centre setting may limit generalisability; (3) the inconsistency between crude and adjusted estimates for amputation underscores the fragility of the observed association; (4) follow - up was not complete for all patients at 3 years, though censoring was handled appropriately; (5) functional outcomes and quality - of - life data were not collected; (6) detailed procedural variables such as contrast volume and thrombolysis protocols were not uniformly recorded. (7) The endovascular treatment group comprised multiple therapeutic modalities, including angioplasty/stenting, catheter-directed thrombolysis, and mechanical thrombectomy. These techniques may be selected according to different thrombotic mechanisms, anatomical patterns, and lesion characteristics. Furthermore, contemporary endovascular practice incorporates evolving technical strategies, including advanced crossing and re-entry techniques and adjunctive intravascular imaging, which were not uniformly captured in our dataset. Therefore, our findings should be interpreted as comparisons between broad treatment strategies rather than evidence supporting the superiority of any specific endovascular technique.

## Conclusion

In this cohort study, endovascular revascularisation for ALLI was associated with improved peri - operative outcomes and a reduced risk of the composite endpoint. Although adjusted analyses demonstrated a lower risk of major amputation with endovascular treatment, this finding was dependent on model specification and contrasted with the higher crude amputation incidence observed in the endovascular group. Therefore, these results should not be interpreted as evidence of superior limb salvage with endovascular treatment. Given the observational design, treatment-selection bias, and potential residual confounding, the observed associations should be considered exploratory and hypothesis-generating rather than causal. Furthermore, the heterogeneity of endovascular techniques included in this cohort limits procedure-specific conclusions. Definitive treatment recommendations await prospective randomised controlled trials.

## Data Availability

The original contributions presented in the study are included in the article/Supplementary Material, further inquiries can be directed to the corresponding author.
