# Supplementary material for: Comparative midterm outcomes of contemporary revascularization strategies for acute lower limb ischemia: a retrospective cohort study
Source: Front Cardiovasc Med. 2026 Sep 29;13:1963564. doi: 10.3389/fcvm.2026.1963564 (PMC13657951; doi:10.3389/fcvm.2026.1963564)
Supplement: Supplementary file 1 [file Datasheet1.docx]

**Supplementary Table S1.** Baseline covariate balance before and after IPTW adjustment

| **Covariate** | **Unweighted SMD** | **Weighted SMD (IPTW)** |
| --- | --- | --- |
| Age | 0.42 | 0.05 |
| Male sex | 0.08 | 0.02 |
| Diabetes mellitus | 0.31 | 0.04 |
| Hypertension | 0.27 | 0.03 |
| Smoking status | 0.33 | 0.06 |
| Dyslipidaemia | 0.29 | 0.05 |
| Atrial fibrillation | 0.36 | 0.07 |
| Chronic kidney disease | 0.41 | 0.06 |
| Rutherford IIb–III | 0.48 | 0.08 |
| Symptom duration (>6h vs ≤6h) | 0.22 | 0.05 |
| Aorto-iliac occlusion | 0.19 | 0.04 |
| Femoro-popliteal occlusion | 0.21 | 0.06 |
| Infra-popliteal occlusion | 0.18 | 0.05 |

**Supplementary Table S2.** Sensitivity analysis excluding Rutherford III ischaemia

| **Outcome** | **Full cohort (n=842)** | **Rutherford III excluded** | **Effect estimate (Endovascular vs Open)** | **95% CI** | **P value** |
| --- | --- | --- | --- | --- | --- |
| Peri-operative mortality | 21.5% | 20.8% | OR 0.34 | 0.16–0.71 | 0.004 |
| Acute kidney injury | 13.2% | 12.6% | OR 0.51 | 0.27–0.96 | 0.034 |
| Major amputation (3-year) | 11.6% / 21.9% / 13.5% | 10.9% / 20.4% / 13.2% | HR 0.74 | 0.53–1.03 | 0.061 |
| All-cause mortality (3-year) | 24.1% / 12.8% / 20.3% | 23.4% / 12.2% / 19.8% | HR 0.90 | 0.62–1.31 | 0.57 |
| Composite outcome (3-year) | 30.5% / 20.1% / 27.4% | 29.8% / 19.6% / 26.9% | HR 0.73 | 0.57–0.94 | 0.012 |
| Re-intervention (3-year) | 10.1% / 21.0% / 18.5% | 9.8% / 20.6% / 18.2% | HR 1.92 | 1.41–2.63 | <0.001 |

**Supplementary Table S3**. Procedural characteristics according to revascularisation strategy

| **Variable** | **Open surgery (n=452)** | **Endovascular (n=198)** | **Hybrid (n=192)** |
| --- | --- | --- | --- |
| Thromboembolectomy (Fogarty catheter) | 312 (69.0%) | — | 118 (61.5%) |
| Bypass surgery | 98 (21.7%) | — | 41 (21.4%) |
| Combined open procedures | 42 (9.3%) | — | 33 (17.2%) |
| Percutaneous transluminal angioplasty (PTA) | — | 162 (81.8%) | 118 (61.5%) |
| Stent implantation | — | 84 (42.4%) | 73 (38.0%) |
| Catheter-directed thrombolysis | — | 66 (33.3%) | 33 (17.2%) |
| Mechanical thrombectomy | — | 59 (29.8%) | 41 (21.4%) |
| Fasciotomy | 76 (16.8%) | 21 (10.6%) | 28 (14.6%) |
| General anesthesia | 401 (88.7%) | 102 (51.5%) | 161 (83.9%) |
| Regional/local anesthesia | 51 (11.3%) | 96 (48.5%) | 31 (16.1%) |
| Aorto-iliac occlusion | 53 (11.7%) | 25 (12.6%) | 25 (13.0%) |
| Femoro-popliteal occlusion | 258 (57.1%) | 112 (56.6%) | 109 (56.8%) |
| Infra-popliteal occlusion | 141 (31.2%) | 61 (30.8%) | 58 (30.2%) |

**Supplementary Table S4.** Propensity score–weighted (IPTW) analysis of outcomes

| Outcome | IPTW-adjusted effect (Endovascular vs Open) | 95% CI | P value |
| --- | --- | --- | --- |
| Peri-operative mortality | OR 0.33 | 0.16–0.70 | 0.004 |
| Acute kidney injury | OR 0.49 | 0.26–0.94 | 0.030 |
| Major amputation (3-year) | HR 0.73 | 0.51–1.04 | 0.078 |
| All-cause mortality (3-year) | HR 0.91 | 0.63–1.33 | 0.62 |
| Composite outcome (death or amputation) | HR 0.70 | 0.55–0.90 | 0.005 |
| Re-intervention (3-year) | HR 1.88 | 1.39–2.55 | <0.001 |
